# The Mechanism of Cumulative Ecological Risk Affecting College Students’ Sense of Social Responsibility: The Double Fugue Effect of Belief in a Just World and Empathy

**DOI:** 10.3390/ijerph20010010

**Published:** 2022-12-20

**Authors:** Yiyu Yi, Qianbao Tan, Jiahui Liu, Fuqun Liang, Chao Liu, Zhenbiao Yin

**Affiliations:** 1 School of Education, Hunan University of Science and Technology, Xiangtan 411201, China; 2 “The 14th Five-Year Plan” Research Base for Education Science, Xiangtan 411201, China; 3 Key Laboratory of Brain, Cognition and Education Sciences, South China Normal University, Ministry of Education, Guangzhou 510631, China; 4 School of Psychology, South China Normal University, Guangzhou 510631, China; 5 School of Computer and Electrical Engineering, Hunan University of Arts and Science, Changde 411500, China; 6 Xiangtan Municipality Public Security Bureau, Xiangtan 411100, China

**Keywords:** cumulative ecological risk, social responsibility, belief in a just world, empathy

## Abstract

According to bioecological theory, the development of college students’ social responsibility is affected by the cumulative effect of ecological risks. However, research on the impact of cumulative ecological risk on social responsibility and its potential mechanisms are still lacking. Carol Gilligan (1982) proposed that the ethics of care and justice are like two related but independent melodies, which together constitute the whole of moral psychology. However, despite being an important part of moral psychology, social responsibility has rarely been investigated empirically with regards to the psychological mechanisms of justice and caring orientation. Therefore, the current study had 1607 college students complete questionnaires regarding cumulative ecological risk, social responsibility, belief in a just world, and empathy, aiming to explore the impact of cumulative ecological risk on college students’ sense of social responsibility and the mediating roles of belief in a just world and empathy. Results showed that: (1) cumulative ecological risk was significantly negatively correlated with college students’ sense of social responsibility, belief in a just world, and empathy, whereas social responsibility, belief in a just world, and empathy were significantly positively correlated; (2) belief in a just world and empathy played mediating roles in the relationship between cumulative ecological risk and social responsibility. The results also showed that the development of college students’ sense of social responsibility was affected by the cumulative ecological risk from various directions; this influence was also seen to play a role in the motivation system of social responsibility through the ethics of care with empathy as the important part, as well as through the ethics of justice. The results suggest that we should reduce the ecological risks at their source, and improve and consolidate students’ social support systems; moreover, we should not only enhance college students’ sense of mission and responsibility to consciously maintain social justice order, but also adopt empathy training as a part of the curriculum to improve students’ empathy at the individual level.

## 1. Introduction

Social responsibility is the spiritual support of human unity that helps us overcome difficulties and move forward courageously. Its development is related not only to individual development, but also to the future and destiny of a nation [1,2]. However, at present, due to a commodity-focused economy, “self-centered” value appropriation, and domination as a common personal aim, goals set in higher education are often driven by instrumental rationality and utilitarianism. Some of the individuals’ sense of social responsibility appears to be weakening gradually, with a growing lack of awareness of political interest, humanistic care, dedication, and environmental issue interest [3,4,5,6,7]. Several issues in this topic have been identified as needing further examination, such as insufficient awareness of personal responsibility, weak will in assuming social responsibility, the trend towards a utilitarian identity, and unrealistic actions undertaken towards fulfilling social responsibility [8,9].

Bioecological theory suggests that each developing individual is in a unique ecological subsystem that interacts with those of others, and the interactions between the individual and the other ecological subsystems shape one’s psychological and behavioral development [10]. As social beings, college students do not exist in a single ecological system, and the formation and development of their sense of social responsibility is not a passive and isolated process, but rather one of dynamic interdependence which mutually influences various environmental factors as students face a variety of risks from the other various sub-ecosystems and fields that they interact with, including those of family, peers, school, and that of the wider community. The risk factors of each ecosystem are not isolated from each other, but rather have strong co-occurrence and interaction [11], and the accumulation of risk factors in various fields can have more serious consequences for individual behavior [12]. Therefore, positioning the cultivation of college students’ social responsibility in the social-ecological system, exploring the relationship between their poor social responsibility development and cumulative ecological risks, and revealing the psychological mechanisms by which cumulative ecological risk factors affect the development of college students’ sense of social responsibility are important to effectively understand and thereby promote its development.

### 1.1. The Relationship between Cumulative Ecological Risk and College Students’ Social Responsibility

Social responsibility refers to the psychological quality of an individual to overcome internal good and evil, resist temptation, proactively comply with social norms, and perform moral duties, taking into account indicators of social responsibility as defined by national consciousness, other individuals, and natural and organizational responsibility [13]. However, the development of individual social responsibility is not always steady, and is affected by both individual and environmental risk factors [14,15]. Cumulative ecological risk asserts that individual development is influenced by multiple risk factors in a variety of contexts, affected by family, school, peers, and community, and that different risk factors will be compounded in the process of affecting individual development; furthermore, the higher the cumulative risk index, the higher the likelihood of individual cognitive, emotional, and behavioral deviation [16].

Negative factors at the environmental level include family risk factors, school risk factors, peer risk factors, and community risk factors [10,17]. In terms of family risk factors, studies have shown that high objective family financial pressure and lack of parental understanding and love are not conducive to the development of a child’s sense of responsibility, and poor parent–child relationships and communication also increase the probability of children using deception to avoid responsibility and punishment [18,19]. With regards to school risk factors, the increased pressure to advance in higher education has led to schools limiting time spent on moral education; however, greater academic pressure, negative school climates, unsupportive teacher leadership, and lower subjective and objective support felt by students in an intensely competitive environment are not conducive to motivating students’ sense of responsibility [20,21]. In terms of peer risk factors, social learning theory emphasizes observational learning, which suggests that individuals observe and imitate the words and behaviors of their peers. The more undesirable peer influence an individual experiences, the more likely they are to avoid moral responsibility [22,23]. In terms of community risk, communities that provide more economic resources, social cohesion, stability, and security, which therefore provide more cognitive stimulation and social interaction, promote steadier personality development [24,25], whereas deprivation of community support is associated with a variety of adverse health and social outcomes [26]. This suggests that risk factors in the growth environment can be a limiting factor in the development of responsibility. Scholars have begun to focus on the role of risk accumulation in various domains on individual development [11,27,28], including the fact that ecological risk factors are gradually being seen as important predictors among the many factors affecting social responsibility. However, there is still a lack of attention paid to risk factors in the development of social responsibility among college students, and a lack of integrated research on risk factors from multiple fields, resulting in a gap in our understanding of effective risk avoidance strategies in the development of social responsibility among college students.

### 1.2. The Mediating Mechanism of Cumulative Ecological Risk Affecting College Students’ Social Responsibility

Damasio considered social responsibility to be a moral psychological quality and investigated its formation process [29], believing that every moral psychological process contains both emotional and rational components. Hoffman also considered empathy and justice as foundations of morality from the perspective of rationality and emotion [30]. In the field of moral psychology, researchers such as Kohlberg have long regarded justice as being a primary motivation of morality. Gilligan (1982) further developed this idea to propose justice ethics and caring ethics, leading to researchers focusing on the relationship between care and justice in a moral context [31]. The relationship between care and justice is not a simple complementary relationship, nor are the two of these diametrically different and opposite orientations or approaches; rather, they represent orientations of human moral development, and the two motivations of moral judgment and moral choice. The differences between them cannot prevent the two from co-existing in the field of moral psychology [32]. These two orientations are like two related but independent themes with fixed harmonious rules, working together as a sort of “double fugue”, forming a harmonious moral psychological process.

To define them more specifically, first, the sense of justice refers to people’s belief that they live in a just world, where people get what they deserve [33]. Numerous studies have demonstrated that a belief in a just world may be the mediating variable between cumulative ecological risk and social responsibility. There is a close negative correlation between ecological risk factors and one’s belief in a just world [34,35,36]. Some studies have pointed out that there is a specific relationship between one’s experience of individual justice and their parental style, and that growing up with strict parenting methods, such as parental rejection and over-protection, is shown to affect children’s perception of social justice [37,38]. As one ages, the influencing factors on an individual’s interpersonal relationship model will gradually transition from family relationships to peer relationships [39]. Power inequalities between peers in the communication process will result in negative impacts on the formation of one’s sense of justice [40], and all existing studies have revealed the negative impact of ecological risk factors on one’s belief in a just world.

Second, one’s belief in a just world has been shown to have a significant positive predictive effect on social responsibility. Some studies have indicated that there is not only a positive correlation between college students’ sense of justice and their sense of social responsibility, but also an obvious causal relationship between the two [41,42]. The higher a college student’s belief in social justice, the stronger their sense of responsibility to actively assume social responsibility or help others [43]. Nudelman and Otto (2021) explored the relationship between one’s sense of justice and having a conscientious personality through meta-analysis, and found that sense of justice is a potential antecedent variable that affects the development of conscientious personality, and that improving one’s sense of personal justice in childhood may promote the development of a conscientious personality in the future [1,44].

The ethic of care is an ethic that “emphasizes interconnectedness, relationships, nurturing, and responsibility towards concrete embodied others” [45]. Ethics of care constitutes a developmental reasoning sequence, from self-concern (Level 1) to caring for others and self-sacrifice (Level 2) to the balanced caring for self and others (Level 3); furthermore, in the order of advancement of these three levels, the care orientation progressively understands moral judgment as adaptive cognition sensitive to the needs of others and the dynamics of specific relationships, and moral reasoning of the care ethics orientation as empathy and concern [46]. Empathy refers to one’s ability to empathize with others in the process of interpersonal communication [47,48]. Empathy is an important concept in the field of care ethics because it is a unique way to connect with others, to understand what is at stake for them, and to help guide moral deliberation [49].

Research has shown that empathy is likely to be strongly related to cumulative ecological risk and social responsibility [50,51,52]. One’s empathy ability can reveal their acceptance of both society and others. A lack of social support from family, school, and peers, however, is not conducive to the development of one’s individual empathy ability [53,54]. Batson and Daniel (1987) put forward the altruistic hypothesis of empathy, which proposes that when others are in trouble, bystanders will experience emotions such as sympathy, compassion, etc. directed towards the victim, and the stronger one’s intensity of experiencing these emotions, the stronger that individual’s motivation to help others solve their difficulties [55]. Namely, the level of one’s empathy reaction can be used to infer how much one relates to the interests of others and wider society in difficult situations, which can make them more likely to notice others’ feelings and needs; meanwhile, a lower level of empathy is not conducive to stimulating one’s altruistic psychological qualities, nor inspiring behaviors such as helping others, cooperating, or donating [56,57]. Social responsibility is also a positive and altruistic psychological quality [13]. Some studies have pointed out that empathy has a significant positive predictive effect on social responsibility [58,59], and that empathy can affect college students’ prosocial behaviors by influencing their sense of social responsibility [60]. It follows, then, that ecological risk factors from family, school, and other areas of one’s life may jointly affect their individual level of empathy, and may further affect the generation of individual social responsibility. However, few studies have explored whether the accumulation of ecological risks affect college students’ sense of social responsibility through empathy.

Therefore, based itself on the findings and theories of the aforementioned existing research, we proposed the first to the fourth hypotheses of this study:

**Hypothesis 1.**  *Cumulative ecological risk is negatively associated with social responsibility, belief in a just world, and empathy; social responsibility, belief in a just world, and empathy are positively associated with each other.*

**Hypothesis 2.**  *Cumulative ecological risk can indirectly affect one’s belief in a just world to negatively predict their sense of social responsibility.*

**Hypothesis 3.**  *Cumulative ecological risk can indirectly negatively affect one’s level of empathy.*

**Hypothesis 4.**  *Cumulative ecological risk can negatively directly affect one’s level of social responsibility, whereas their belief in a just world and level of empathy play parallel mediating roles in the relationship between cumulative ecological risk and social responsibility.*

To summarize, when faced with a myriad of complicated moral choices and situations, and by considering the ethics of care and justice as important motivators in the relationship between empathy and systematic justice, it may be possible to better enhance one’s sense of social responsibility. Therefore, this study explored whether cumulative ecological risk affects college students’ sense of social responsibility, and how that cumulative ecological risk affects the development of college students’ sense of social responsibility. Based on the existing research, we introduced mediating variables (i.e., belief in a just world and empathy) while also drawing on bioecology theory to explore the impact of “justice” (belief in a just world) and “care” (empathy) on the development of college students’ sense of social responsibility, with a view to further reveal the psychological mechanism of cumulative ecological risk factors affecting college students’ sense of social responsibility.

## 2. Material and Methods

### 2.1. Participants and Procedure

This study focused on Chinese college students. A total of 1632 valid questionnaires were collected from six different universities from across China. After excluding respondents who did not respond to items regarding key variables such as social responsibility, 1607 questionnaires were ultimately included in the analysis. The total comprised 690 males and 917 females, with 442 freshmen, 435 sophomores, 370 juniors, and 360 seniors (*M*_age_ = 20.35, *SD*_age_ = 1.51, age ranging from 17 to 26 years old). The survey data was collected in May 2021 by teachers and graduate students trained by the study researchers. Before conducting this study, we obtained ethical approval from the Institutional Review Board (IRB) of Hunan University of Science and Technology. After the researchers obtained permission to carry out the study from the school management, all college students gave their written informed consent after being told the purpose and procedures of the study and before they began to respond to the questionnaires. Participants were told that their participation was entirely voluntary and that they could withdraw from the survey at any time. The same written instructions were given before each survey was distributed, and the survey was completed in a classroom setting during normal class hours, and were collected immediately upon completion of the survey.

### 2.2. Measures

Measurement of Risk Factors and Construction of Cumulative Risk Index.

The independent variable of this study was cumulative ecological risk. The Cumulative Risk Index Questionnaire included 11 indicators; variables and descriptions of all are shown in Table 1. Following previous research, the risk factor score was binary coded, with the 25th or 75th quantile of the risk factor score taken as the risk threshold, 1 representing risk, and 0 representing no risk. The total score was obtained by adding together the risk factors after each coding, that is, determining the cumulative ecological risk index. With regards to the “community security risk factor” index, a score of less than 2 was coded as 1, and a score of 3 or more was coded as 0 [16,28]. Specific risk factors are shown in Table 1.

Objective socioeconomic status. The questionnaire compiled by Zhou and Guo (2013) was used to measure the parental education level according to China’s national educational levels [19]. There were two scales, and each scale was divided into six levels: primary school and below, junior high school, high school/technical secondary school, college, undergraduate, and Master’s degree and above. These six levels were assigned 1 to 6 points, respectively. Following Lu (2003) [61], parents’ occupation was measured on one dimension divided into 10 levels: urban and rural unemployed and semi unemployed, agricultural workers, industrial workers, commercial service workers, self-employed businesses, clerks, professional technicians, private entrepreneurs, managers, and state and social managers. Each level was assigned from 1 to 10 points, respectively. Annual household income was measured using the questionnaire developed by Li et al. (2019) [62]. This measure was divided into 10 levels, with the lowest being less than RMB 1000 per year (equivalent to USD 140/year) and the highest being more than RMB 1 million per year (equivalent to USD 140,000/year). After determining these three values, the three items were calculated. First, the higher scores in occupational grade and education level between the father and mother was chosen for the calculation. The five indicators were combined to make three indicators: parents’ occupations, parents’ level of education, and annual family income. These three indicators were then converted into standard scores, and the principal component analysis method was used for the standard scores. The final calculation formula was: Objective SES = (0.83× *Z* occupation grade + 0.80 × *Z* education level + 0.57 × *Z* family annual income)/1.65. The factor loading of each corresponding *Z*-variable was 0.83, 0.80, and 0.57, respectively, and the characteristic value of the first factor was 1.65. The higher the score, the higher the objective SES score, indicating that the family had a higher objective economic and social status.

Subjective Socioeconomic Status. We used the one relevant item from the MacArthur 10-Step Scale [63], which had a total possible score of 10 points. The scale uses a ladder which respondents use to represent their family’s social position from low (1) to high (10). The higher the level, the higher their family’s perceived social status.

Family Relationships. The study used the Family Intimacy and Adaptability Scale, as compiled by Olson (2000) and adapted by Xu et al. (2008) [64,65]. The scale uses 20 items in total, and participants are required to rate each item on a scale from 1 (never) to 5 (always). The higher the score, the higher the family cohesion and adaptability. In this study, the Cronbach’s alpha coefficient of the scale was 0.93.

Family Support. The study used the family support dimension from the Perceived Social Support Scale as developed by Zimet et al. (1988) and adapted by Yan and Zhen (2006) [66,67]. The scale uses four items in total, with participants rating each item on a scale from 1 (strongly disagree) to 7 (strongly agree). The higher the score, the stronger the respondent’s perceived family support. In this study, the Cronbach’s alpha coefficient for the scale was 0.89.

School Connectivity. The School Connectedness Scale was developed by Resnick et al. (1997) and translated by Yu et al. (2011) [68,69], and includes six items in total. Participants rate each item from 1 (strongly disagree) to 5 (strongly agree). The higher the score, the higher the individual’s perceived school connectedness. In this study, the Cronbach’s alpha coefficient of the scale was 0.90.

Teacher Leadership. The study used the Dimensions of Transformational Leadership and Transactional Leadership Scale adopted from the Multifactorial Leadership Questionnaire as developed by Bass (1981) and Luo (2012) [70,71]. The scale includes 18 items, and participants rate each item on a scale from 1 (never) to 5 (often). The higher the score, the more the teacher’s leadership style conforms to the transformational leadership or transactional leadership styles. In this study, the Cronbach’s alpha coefficient of the scale was 0.97.

Teacher Support. Items from the teacher support dimension from the Adolescents’ Perception of School Atmosphere Measure developed by Jia et al. (2009) [72] were used to assess teacher support. The dimension comprises seven items, and participants rate each item from 1 (never) to 4 (always). The higher the total score, the more teacher support is perceived by the respondent. In this study, the Cronbach’s alpha coefficient of the scale was 0.81.

Friend Support. The Perceived Social Support Scale, as developed by Zimet et al. (1988) and adapted by Yan et al. (2006) [66,67], was used to assess the friend support dimension. The scale comprises four items, and participants rate each item on a scale from 1 (strongly disagree) to 7 (strongly agree). The higher the score, the more support from friends is perceived by the respondent. In this study, the Cronbach’s alpha coefficient of the scale was 0.90.

Peer Alienation. The peer alienation dimension was adopted from the Parental and Peer Attachment Questionnaire compiled by Armsten and Greenberg (1987) and adapted by Song (2004) [73,74]. The scale includes seven items in total, with participants rating each item from 1 (never) to 5 (always). The higher the total score, the more alienated the respondent feels in terms of their peer relationships. The Cronbach’s alpha coefficient of the scale in this study was 0.79.

Community Security. Following Gerard and Buehler (2004) [47], one item was used to evaluate respondents’ overall sense of community security. Participants were asked to rate the item on a scale ranging from 1 (very unsafe) to 4 (very safe). The lower the score, the less safe the individual feels in their community environment.

Neighborhood Support. The current study used the measure compiled by Sun et al. (2021) [28] to assess neighborhood support. It uses two items, and each one is scored on a scale ranging from 1 (no neighbors around) to 4 (very familiar). The lower the score, the less the respondent perceives support within their neighborhood.

Social Responsibility. The dependent variable of this study was social responsibility. To measure this, we used the Social Responsibility Scale compiled by Liu et al. (2017) [13], which includes four dimensions: national responsibility, natural responsibility, others’ responsibility, and organizational responsibility. The scale includes 17 items in total, with each item scored on a scale ranging from 1 (never) to 5 (always). The higher the total score, the stronger the respondent’s sense of social responsibility. The Cronbach’s alpha coefficient of the scale in the current study was 0.95.

Belief in a Just World. The Global Just World Belief Scale developed by Brandon et al. (2015) [75] was used to measure the Belief in a Just World variable in this study. It includes seven items, with each item rated on a scale from from 1 (strongly disagree) to 7 (strongly agree). The higher the total score, the higher one’s belief in a just world. The Cronbach’s alpha coefficient in the current study was 0.89.

Empathy. Empathy was measured using the Interpersonal Reactivity Index [76]. The scale measures four dimensions: opinion selection, empathy, fantasy, and personal pain. It comprises 28 items, and each item is rated on a scale ranging from 1 (strongly disagree) to 5 (strongly agree). The higher the total score, the higher one’s level of empathy. The Cronbach’s alpha coefficient of the empathy questionnaire in this study was 0.80.

Demographic Variables. Previous studies have found that gender and school grade may impact the development of one’s level of individual responsibility [77,78]. Therefore, this study considered both gender and college year of subjects as control variables.

### 2.3. Data Analysis

The survey was conducted by a professionally trained teacher and a graduate student in the students’ classroom during normal school hours. The group test was conducted using uniform guidelines, and the survey was completed immediately after finishing the test. SPSS Statistics 21.0 was used for the reliability test, the Harman common method deviation test, descriptive statistical analysis, and correlational analysis using the general linear regression method. AMOS 24.0 and MPLUS 8.0 were used for mediating model testing.

## 3. Results Analysis

### 3.1. Common Method Deviation Test

Self-reporting can lead to common methodological bias in research. Therefore, we controlled for this by using reverse scoring on some items and ensuring that the surveys and research tools were kept anonymous, as well as using the Harman single factor test to check for common method bias effect in the analysis data [79]. The results showed that the variance explained by the first main factor was 16.78%, which did not reach the critical value of 40%, indicating that the common method deviation was not obvious in this study.

### 3.2. Descriptive Statistics

#### Hypothesis 1: Cumulative Ecological Risk Is Negatively Associated with Social Responsibility, Belief in a Just World, and Empathy; Social Responsibility, Belief in a Just World, and Empathy Are Positively Associated with Each Other

Means, standard deviations, and correlation coefficients of the main variables are shown in Table 2. Results of the analysis showed that cumulative ecological risk was negatively correlated with social responsibility, belief in a just world, and empathy (*p* < 0.01). There was a significant positive correlation between social responsibility, belief in a just world, and empathy (*p* < 0.01).

An independent sample *t*-test was conducted on gender differences in cumulative ecological risk and social responsibility. The results showed that the cumulative ecological risk score reported by girls was significantly lower than that reported by boys: *t* (916) = 2.47, *p* < 0.05, *d* = 0.12. The gender difference in social responsibility was not significant (*p* = 0.06). Single factor ANOVA was used to analyze grade differences on cumulative ecological risk and social responsibility. The results showed that there were no significant differences in cumulative ecological risk (*p* = 0.15) and social responsibility (*p* = 0.87) according to grade. Therefore, we used only gender as a control variable in the mediation analysis.

### 3.3. Influence of Ecological Risks Indices and Risk Fields on Social Responsibility

As strong data conclusions could not be drawn from the binary correlation coefficient results in the correlation analysis of the variables in our initial findings, the questionnaire data was analyzed further using the general linear regression method in SPSS Statistics 21.0. In Model 1, we regarded social responsibility as the dependent variable, with the ecological risk indices as the independent variables and gender as the control variable. In Model 2, we regarded social responsibility as the dependent variable, with risk fields as the independent variables and gender as the control variable.

As shown in Table 3, in Model 1, from the perspective of ecological risks indices, family relationships, family support, school connection, teacher leadership style, friend support, community safety, and neighborhood support risks were all shown to negatively predict college students’ social responsibility (*p* < 0.05) or (*p* < 0.01), with family relationships and teacher leadership style playing greater roles in influencing college students’ social responsibility. In Model 2, from the perspective of risk fields, risks from family, school, peer, and community fields were shown to negatively predict college students’ social responsibility (*p* < 0.01), with risks from the school and family fields playing greater roles in influencing college students’ social responsibility.

### 3.4. Detection of the Mediating Effects of Belief in a Just World and Empathy

#### 3.4.1. Hypothesis 2: Cumulative Ecological Risk Can Indirectly Affect One’s Belief in a Just World to Negatively Predict Their Sense of Social Responsibility

**Figure 1:**
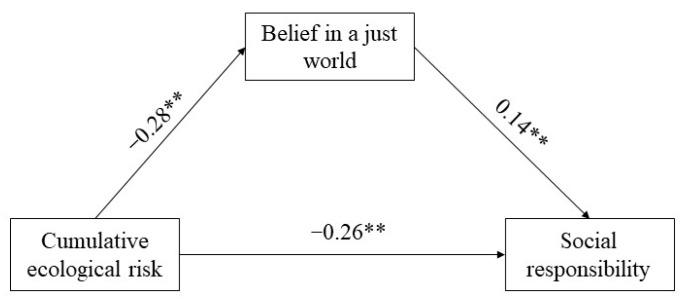
Mediating Model of Belief in a Just World. Notes. ** *p* < 0.01.

To test Hypothesis 2, we used social responsibility as the dependent variable, cumulative ecological risk as the independent variable, belief in a just world as the mediating variable, and gender as the control variable, standardizing and testing the mediating effect using the bootstrap method. The results showed that cumulative ecological risk had a significant negative predictive effect on college students’ sense of social responsibility (*β* = −0.30, *p* < 0.01). When the mediating variables were entered into the model, cumulative ecological risk continued to significantly negatively predict social responsibility (*β* = −0.26, *p* < 0.01). Cumulative ecological risk also significantly negatively predicted belief in a just world (*β* = −0.28, *p* < 0.01), and belief in a just world significantly predicted social responsibility (*β* = 0.14, *p* < 0.01). We then tested the mediating effect of belief in a just world, and the results showed that the direct effect accounted for 86.67% of the total effect (*β* = −0.26, 95% CI = [−0.31, −0.22]), and the indirect effects accounted for 13.33% of the total effects (*β* = −0.04, 95% CI = [−0.06, −0.02]). The results showed a good model fit, and that cumulative ecological risk not only directly and negatively predicted social responsibility, but also negatively predicted social responsibility through belief in a just world. The model is shown in Figure 1: *χ*^2^ = 6.351, *df* = 1, *χ*^2^/*df* = 6.35, *p* < 0.05, CFI = 0.98, TLI = 0.90, RMR = 0.01, GFI = 0.99, RMSEA = 0.06.

#### 3.4.2. Hypothesis 3: Cumulative Ecological Risk Can Indirectly and Negatively Affect One’s Level of Empathy

**Figure 2:**
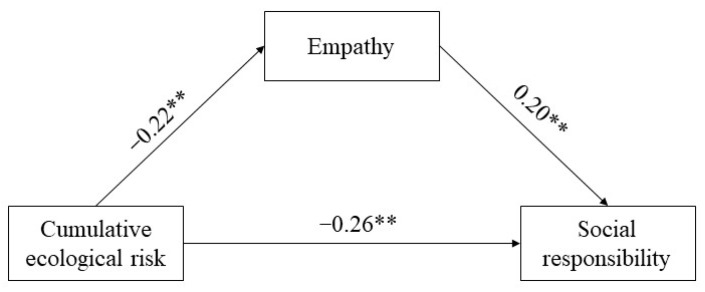
Mediating Model of Empathy. Notes. ** *p* < 0.01.

To test Hypothesis 3, we set social responsibility as the dependent variable, cumulative ecological risk as the independent variable, empathy as the mediating variable, and gender as the control variable. After standardizing the data, mediating analysis was conducted using the bootstrap method. The results showed that cumulative ecological risk had a significant negative predictive effect on college students’ sense of social responsibility (*β* = −0.30, *p* < 0.01). When the mediating variable was entered into the model, cumulative ecological risk still significantly negatively predicted social responsibility (*β* = −0.26, *p* < 0.01) as well as empathy (*β* = −0.22, *p* < 0.01), and empathy significantly predicted social responsibility (*β* = 0.20, *p* < 0.01). We then examined the mediation effect of empathy. The results showed that the direct effect accounted for 86.67% of the total effect (*β* = −0.26, 95% CI = [−0.30, −0.22]), and indirect effects account for 13.33% of the total effects (*β* = −0.04, 95% CI = [−0.06, −0.03]). This result shows that cumulative ecological risk not only directly and negatively predicted social responsibility, but also negatively predicted empathy, thereby negatively predicting social responsibility. The model fit well, and is shown in Figure 2: *χ*^2^ = 6.35, *df* = 1, *χ*^2^/*df* = 6.35, *p* < 0.05, CFI = 0.98, TLI = 0.90, RMR = 0.01, GFI = 0.99, RMSEA = 0.06.

#### 3.4.3. Hypothesis 4: Cumulative Ecological Risk Can Negatively and Directly Affect One’s Level of Social Responsibility, Whereas Their Belief in a Just World and Level of Empathy Play Parallel Mediating Roles in the Relationship between Cumulative Ecological Risk and Social Responsibility

**Figure 3:**
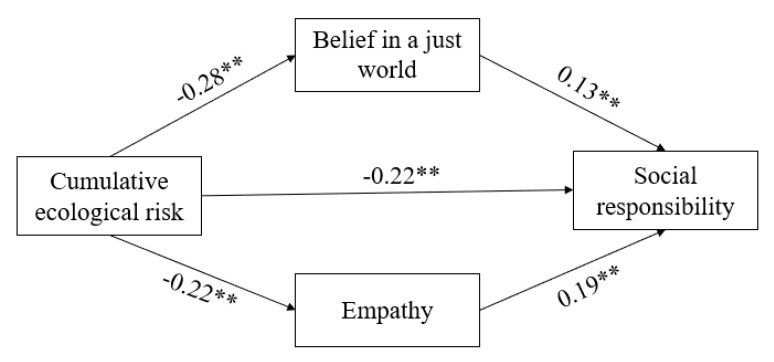
Explicit Mediating Model of Belief in a Just World and Empathy. Notes. ** *p* < 0.01.

Both belief in a just world and empathy are mediating variables in how cumulative ecological risk affects social responsibility. Therefore, following our Hypothesis 4, our next step was to use social responsibility as the dependent variable, with cumulative ecological risk as the independent variable, belief in a just world and empathy as the two mediating variables, and gender as the control variable to conduct a parallel mediation analysis using the bootstrap method. As shown in Table 4, cumulative ecological risk still significantly and negatively predicted social responsibility (*β* = −0.22, *p* < 0.01). Meanwhile, cumulative ecological risk not only significantly predicted one’s belief in a just world (*β* = −0.28, *p* < 0.01), but also significantly and positively predicted social responsibility (*β* = 0.13, *p* < 0.01). Cumulative ecological risk also significantly and negatively predicted empathy (*β* = −0.22, *p* < 0.01), while also significantly and positively predicting social responsibility (*β* = 0.19, *p* < 0.01). Direct effects accounted for 68.75% of the total effects (*β* = −0.22, 95% CI = [−0.27, −0.18]). Meanwhile, the indirect effect mediated by belief in a just world accounted for 12.50% of the total effect (*β* = −0.04, 95% CI = [−0.06, −0.02]), whereas indirect effects mediated by empathy accounted for 12.50% of the total effects (*β* = −0.04, 95% CI = [−0.06, −0.03]). The results showed that the model fit well, and the model can be seen in Figure 3: *χ*^2^ = 8.58, *df* = 2, *χ*^2^/*df* = 4.29, *p* < 0.05, CFI = 0.98, TLI = 0.94, RMR = 0.01, GFI = 0.99, RMSEA = 0.05.

Overall, the best fitting model included both belief in a just world and empathy. Cumulative ecological risk not only directly and negatively affected college students’ sense of social responsibility, but also indirectly and negatively affected their level of social responsibility through the two paths of belief in a just world and empathy. Furthermore, by subtracting the effects of the two indirect paths, no significant difference was found between the indirect path mediated by belief in a just world and the indirect path mediated by empathy (*d* = 0.01, *p* = 0.57).

**Figure 4:**
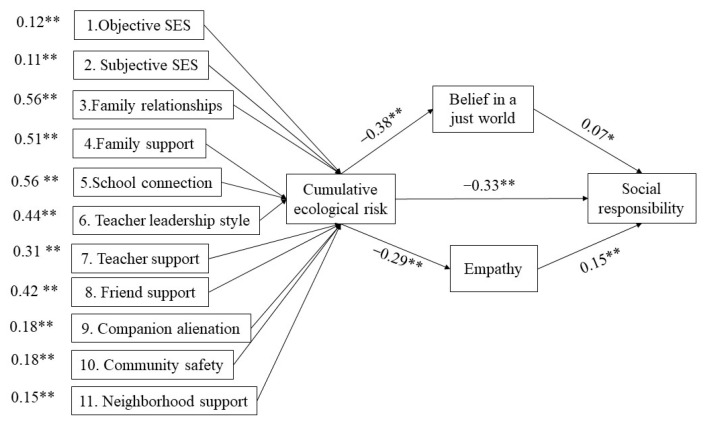
Latent Variable Structural Equation Mediating Model of Belief in a Just World and Empathy. Notes. * *p* < 0.05, ** *p* < 0.01.

MPLUS 8.0 was used to conduct the multiple mediated effects analysis of the latent variable structural equation model to establish the cumulative ecological risk–social responsibility model as mediated by belief in a just world and empathy. Here, cumulative ecological risk was a latent variable with 11 risk indices, and gender was the control variable. The model fit was estimated using the maximum likelihood method, and the fit indicators were only average (*χ*^2^ = 566.03, *df* = 86, *χ*^2^/*df* = 6.58, *p* < 0.01, CFI = 0.76, TLI = 0.71, RMR = 0.01, SRMR = 0.05, RMSEA = 0.06), which showed that the model fit was unacceptable. The results of the multiple mediated effects model analysis are shown in Figure 4.

## 4. Discussion

The current study identified some representative factors with which to construct the cumulative ecological risk index, and used 1607 college students as subjects to explore the impact of cumulative ecological risk on college students’ sense of social responsibility and its internal mechanisms. The study found that cumulative ecological risk showed a significantly negative correlation with belief in a just world, empathy, and social responsibility, whereas positive correlations were found between the same three variables. Furthermore, cumulative ecological risk not only directly and negatively predicted college students’ social responsibility, but also negatively affected college students’ belief in a just world, and thus their sense of social responsibility, as well as negatively affecting their level of empathy, and thus their sense of social responsibility.

### 4.1. The Effect of Cumulative Ecological Risks on College Students’ Sense of Social Responsibility

Cumulative ecological risk can directly predict one’s sense of social responsibility in college students; that is, the higher their cumulative risk index, the lower the development of college students’ sense of social responsibility. In addition, family relationships and teacher leadership style played greater roles in influencing college students’ social responsibility; as for these four risks fields, risks from the school and family fields played greater roles in influencing college students’ social responsibility. This finding is partially consistent with the theory of bioecology which suggests that a barren social environment can aggravate immoral behaviors [18,80]. The reason for this is that college students’ relationships are inevitably affected by risk factors from multiple ecological fields, and risk factors from various fields will all have an important impact on the cultivation of one’s development of their own internal moral responsibility. Family factors, poor family economic conditions, estranged family relationships, limited parental emotional support, and a focus on utilitarianism in particular can all make it difficult for college students to cope with life, despite support from other aspects of society [81]. In terms of school factors, focusing on building knowledge merely for quick success and immediate benefit without spending sufficient time on ideological and moral education, has a negative impact on students’ development of a sense of social responsibility [82]. Moreover, teachers’ passion for teaching and belief in one’s responsibility as a teacher has become progressively challenged in a number of ways, including the fact that the college sector is increasingly in favor of more rigid forms of management [83], leading to teachers investing less care in the personal development of their students either through teaching or disciplinary actions. This can increasingly lead students to feel disconnected and even helpless, resulting in even less development of students’ sense of responsibility [84]. With regards to peer factors, after entering university, the new diversity of one’s peers can increase the potential for friction between students’ living habits and values. Unharmonious peer relationships can result in college students seeking out less peer support, leading to an increased sense of loneliness [85,86]. Ultimately, it is clear that risk factors from all various ecological fields have strong impacts on college students’ sense of social responsibility individually, but even more so when taken altogether, and these risk factors usually compound upon one another in their impact [28,87], leading to a negative correlation between cumulative ecological risk and college students’ sense of social responsibility.

### 4.2. Analysis of the Mediating Role of the Belief in a Just World and Empathy

The current study found that cumulative ecological risk not only directly and negatively predicted college students’ sense of social responsibility, but also affected their sense of social responsibility through their belief in a just world. This finding is somewhat consistent with our Hypothesis 2 regarding the motivation theory of social responsibility [88]. For individuals to consciously perform their socially-expected duties and obligations, they must be motivated by the fairness of the existing social rules and positive codes of conduct specific to their environment [1]. Belief in a just world serves two functions. First, it provides a meaningful framework of understanding and offers strategies, which can help people deal with the threat of unfair events, allowing them to establish a sense of control over their world, and leading them to willingly follow moral principles and social norms to rebuild a sense of justice on a realistic or cognitive level when their morals are threatened. Second, as a personal resource or psychological buffer, belief in a just world can reduce the negative impact of unfair events, helping one maintain positive mental health and providing them with the motivation to pursue long-term goals [89,90]. However, the combination of discrimination or indifference from family, school, peers, or communities and a lower level of social trust can lead to college students experiencing severe adversity, causing them to generally have a lower willingness to cooperate and share with others, which can weaken their receptiveness to the guiding roles that the immediate social justice value system and behavioral norms should play on their individual sense of social responsibility [91].

### 4.3. Analysis of the Mediating Role of Empathy

Cumulative ecological risk not only directly and negatively predicted college students’ sense of social responsibility in the current study, but it also affected their sense of social responsibility through empathy. This finding is partially consistent with Hypothesis 3 regarding the altruistic hypothesis of empathy [55]. Although empathy is inherent to all individuals, one’s individual level of empathy is affected by many factors. For example, a lack of developmental support from family, a lack of educational resources from schools, low recognition of the cultural atmosphere on university campuses, and experiencing indifference in teacher–student or peer relationships can all hinder the development of one’s emotional awareness and expression, particularly in high-risk individuals, thus reducing their level of empathy [57,92,93]. At the same time, empathy plays a very important role in social interaction, promoting the development of sound psychological strength and responsible behavior [94]. Individuals with low empathy are more inclined to look for excuses to escape social responsibility [95,96]. Therefore, as the cumulative number of risk factors from family, school, peers, and other areas increases, the degree of one’s internal spiritual friction deepens, and college students lose their ability to experience empathy, greatly reducing their sense of responsibility, emotional breadth, and behavioral awareness, thus reducing their level of individual social responsibility.

### 4.4. Analysis of the Mediating Role of the Belief in a Just World and Empathy

Finally, cumulative ecological risk was shown to negatively affect the level of college students’ social responsibility both directly and indirectly through their belief in a just world and empathy together, where belief in a just world and empathy play parallel mediating roles in the relationship between cumulative ecological risk and social responsibility. This finding was partially consistent with Hypothesis 4 and Carol Gilligan’s (1988) proposal that caring ethics and justice ethics are the two moral orientations inherent to understanding moral psychology, working as independent yet related variables [97,98]. Moral judgment involves empathy and fairness, both of which are related to the anterior insula cortex, and are key to altruism and, and thus the two are positively closely related; for example, we generally empathize more with people we feel close to or people who we think are more fair [99], or we perceive fair people as being more compassionate [100]. However, these two systems may also disagree or interfere with one another when one is assessing a given situation [29]. Batson et al. (1995) suggested that individuals act based on justice when empathy and justice are in conflict, but if they are stimulated to feel empathy, they should engage in altruistic behavior based on empathy [101]. For example, in a moral allocation dilemma, individuals’ moral identity increases donations for recipients with low plight responsibility (massive layoffs during economic recession) through increased empathy and decreased justice; however, individuals’ moral identity decreases donations to recipients with high plight responsibility (drug or alcohol abuse) due to more perceptions of justice and less perceptions of empathy [102]. According to Greene et al. (2004) [103], empathy is more engaged in more personal situations and tends to be more of a deontological judgment, whereas justice works better in more impersonal situations and tends to be more of a utilitarian judgment [29]. In daily life, both the ethics of justice with core fairness and the justice and ethics of care with empathy as the important part are essential, and they influence and interpenetrate each other, so that an individual can expand the circle of ethical concerns and become more balanced between empathic and fair in the constant game of moral psychology, which is more conducive to one’s social responsibility development [104].

## 5. Implications and Limitations

Therefore, to cultivate college students’ sense of social responsibility, we should reduce ecological risk factors that negatively affect its development, while improving and strengthening college students’ social support systems. In terms of family support, parents should avoid both excessively doting on their children without tending towards neglect, and instead strengthen their emotional support and educate their children regarding responsibility while creating a harmonious and supportive family atmosphere. In terms of the study environment, colleges and universities should limit focus on utilitarianism in education and teaching styles, and instead attach more importance to the social service functions of college and university while strengthening their moral care for students. In terms of community support, social service institutions should strengthen their supervision of malpractice, publicize aspirational stories of conscientiousness and responsible actions, and focus on building a good societal sense of morality.

Meanwhile, in consideration of both moral orientations of justice and care, we should focus on cultivating college students’ abilities in empathy and their belief in a just world, encouraging college students to internalize care and justice. First, we should optimize individuals’ internal sense of justice in order to enhance college students’ motivation and personal responsibility in consciously maintaining social justice. Second, we should introduce training in empathy to improve college students’ empathetic abilities and, therefore, strengthen their ability to resist the negative impacts of cumulative ecological risks on their sense of social responsibility.

Furthermore, the COVID-19 century pandemic not only threatened global public health, but also brought a heavy hit to the economic development, and individuals had to face more risk factors, which indicates in the future, a great number of nations are interlocked, and the fight against the pandemic is the responsibility of the whole society. Under the influence of the pandemic, governments not only pursue fairness and justice in the distribution of food and medicine, but also express humanistic care to people in the infected areas. A caring and just society is more likely to awaken people’s sense of social responsibility, emotions and behaviors, and is more likely to make the international community launch more beneficial policies to deal with this major global crisis.

We must acknowledge that there are still some limitations to this study. First, the nature of our study design was cross-sectional, which makes it difficult to draw causality inferences and deeply explore the development of college students’ sense of social responsibility and the dynamic process of ecological risk. Future research should employ more longitudinal or experimental study designs to investigate and discover the development of individual social responsibility and its changing characteristics. Second, the variables assessed were limited to self-reporting responses. Future study should draw on multiple sources of data (such as parents, peers, and teachers) to avoid common method bias as much as possible. Finally, this study was based on a sample of 1607 Chinese college students and thus it would be relatively conservative to extend the results to other age groups or occupational groups, or to populations outside of China. Future research should verify the conclusions of this study among teenagers or middle-aged adults who are also exposed to various risks, or explore whether there are intercultural differences beyond cultural background.

## 6. Conclusions

Rooting itself in the theories of bioecology, responsibility motivation, and empathy and prosocial behavior, this study looked at ecological risk as a threat to the basic life needs of college students, specifically with regards to moral care and justice orientations. The study showed that justice and empathy are important mediating variables through which cumulative ecological risk affects college students’ sense of social responsibility. Furthermore, results showed that mature college students with internalized morality rely on caring ethics with empathy as important, and on justice ethics with belief in a just world as the core in the motivation of their understanding of social responsibility, and these two moral motivations are independent yet related variables, with both affected by family, school, peer, community, and other social factors in their growth process, all of which can affect the development of their sense of social responsibility.

## Figures and Tables

**Table 1:** Descriptions and Definitions of Cumulative Ecological Risk Questionnaire.

Risk Fields	Cumulative Risk Index	Number of Items	Ecological Risk Definition Standard	Proportion of Risk Population
Family	1. Objective SES	5	Below 25th percentile	26.10%
	2. Subjective SES	1	Below 25th percentile	25.30%
	3. Family relationships	20	Below 25th percentile	26.10%
	4. Family support	4	Below 25th percentile	27.90%
School	5. School connection	6	Below 25th percentile	22.50%
	6. Teacher leadership style	18	Below 25th percentile	25.60%
	7. Teacher support	7	Below 25th percentile	18.50%
Peers	8. Friend support	4	Below 25th percentile	26.80%
	9. Companion alienation	7	Below 75th percentile	27.10%
Community	10. Community safety	1	Less than 3	59.40%
	11. Neighborhood support	2	Below 75th percentile	44.80%

Notes. Objective SES = Objective socioeconomic status, Subjective SES = Subjective socioeconomic status.

**Table 2:** Correlation of Means, Standard Deviations, and Study Variables (N = 1607).

Variables	M	SD	1	2	3	4	5	6
1. Gender	1.57	0.50	_					
2. Grade	2.40	1.11	−0.14 **	_				
3. Cumulative ecological risk	3.29	2.10	−0.06 *	0.02	_			
4. Social responsibility	3.91	0.72	0.05	−0.02	−0.30 **	_		
5. Belief in a just world	4.92	1.08	0.07 **	0.06 *	−0.28 **	0.21 **	_	
6. Empathy	3.40	0.36	0.21 **	−0.14 **	−0.23 **	0.26 **	0.11 **	_

Notes. * *p* < 0.05, ** *p* < 0.01.

**Table 3:** Influence of Ecological Risks and Risk Fields on Social Responsibility.

Model 1	*β*	*SE*	*χ* ^2^	*t*	Model 2	*β*	*SE*	*χ* ^2^	*t*
Constants	4.21	0.03		122.28 **	Constants	4.24	0.03		125.07 **
Objective SES	−0.01	0.04	−0.01	−0.22	Family	−0.08	0.02	−0.12	−4.90 **
Subjective SES	0.03	0.04	0.02	0.77					
Family relationships	−0.21	0.04	−0.13	−4.74 **					
Family support	−0.14	0.04	−0.09	−3.23 **					
School connection	−0.11	0.05	−0.06	−2.33 *	School	−0.15	0.02	−0.19	−7.60 **
Teacher leadership style	−0.18	0.04	−0.11	−4.35 **					
Teacher support	−0.08	0.05	−0.04	−1.81					
Friend support	−0.12	0.04	−0.07	−2.95 **	Peer	−0.09	0.03	−0.08	−3.33 **
Companion alienation	−0.04	0.04	−0.02	−0.95					
Community safety	−0.09	0.04	−0.06	−2.45 *	Community	−0.09	0.02	−0.10	−4.23 **
Neighborhood support	−0.09	0.04	−0.06	−2.41 *					

Notes. * *p* < 0.05, ** *p* < 0.01.

**Table 4:** Analysis of the Mediating Effects of the Belief in a Just World and Empathy (*N* = 1607).

Path	Estimate	SE	Bootstrapping		*p*	Bootstrapping		*p*
			Bias-Corrected 95% CI			Percentile 95% CI		
			Lower	Upper		Lower	Upper	
Total effect	−0.30	0.02	−0.34	−0.26	<0.01	−0.34	−0.26	<0.01
CR-SR	−0.22	0.02	−0.27	−0.18	<0.01	−0.27	−0.18	<0.01
CR-Belief in a just world-SR	−0.04	0.01	−0.06	−0.02	<0.01	−0.05	−0.02	<0.01
CR-Empathy-SR	−0.04	0.01	−0.06	−0.03	<0.01	−0.06	−0.03	<0.01
Mediation path difference	0.01	0.01	−0.02	0.03	0.62	−0.02	0.03	0.57

Notes. CR = Cumulative ecological risk, SR = Social responsibility.

## Data Availability

Data may be provided on a reasonable request by contacting the corresponding authors of our study.
